# Defenses Against ROS in Crops and Weeds: The Effects of Interference and Herbicides

**DOI:** 10.3390/ijms20051086

**Published:** 2019-03-02

**Authors:** Andréia Caverzan, Cristiano Piasecki, Geraldo Chavarria, C. Neal Stewart, Leandro Vargas

**Affiliations:** 1 Faculty of Agronomy and Veterinary Medicine, Agronomy Post-Graduate Program, University of Passo Fundo (UPF), Passo Fundo 99052-900, Brazil; acaverzan@hotmail.com (A.C.); geraldochavarria@upf.br (G.C.); 2 Department of Crop Protection, Federal University of Pelotas, Pelotas 96160-000, Brazil; 3 Department of Plant Sciences, University of Tennessee, Knoxville, TN 37996-4561, USA; nealstewart@utk.edu; 4 Department of Weed Science, Brazilian Agricultural Research Corporation (EMBRAPA), Passo Fundo 99050-970, Brazil; leandro.vargas@embrapa.br

**Keywords:** reactive oxygen species (ROS), oxidative stress, herbicide treatment, herbicide resistance, weed evolution

## Abstract

The antioxidant defense system acts to maintain the equilibrium between the production of reactive oxygen species (ROS) and the elimination of toxic levels of ROS in plants. Overproduction and accumulation of ROS results in metabolic disorders and can lead to the oxidative destruction of the cell. Several stress factors cause ROS overproduction and trigger oxidative stress in crops and weeds. Recently, the involvement of the antioxidant system in weed interference and herbicide treatment in crops and weeds has been the subject of investigation. In this review, we address ROS production and plant mechanisms of defense, alterations in the antioxidant system at transcriptional and enzymatic levels in crops induced by weed interference, and herbicide exposure in crops and weeds. We also describe the mechanisms of action in herbicides that lead to ROS generation in target plants. Lastly, we discuss the relations between antioxidant systems and weed biology and evolution, as well as the interactive effects of herbicide treatment on these factors.

## 1. Introduction

In the field, plant growth, development, and reproduction are affected to various degrees by biotic and abiotic factors. Plant stress is most often the result of combinations of biotic and abiotic factors; interactions are complex but need to be understood since they affect plant performance [1]. Stress conditions often induce the overproduction of reactive oxygen species (ROS), which are toxic molecules that can lead to the oxidative destruction of cells. Plants have cellular antioxidant machinery, including enzymatic and non-enzymatic antioxidants that control ROS levels and maintain cellular homeostasis [2,3,4].

The enzymatic and non-enzymatic responses to abiotic stress are widely studied in crops. One gap in our knowledge is the dynamic effects of weed interference on crops and changes rendered by herbicide exposure, especially at the molecular level. Antioxidant response to stress caused by weed interference and herbicide treatment in crops and weeds have been recent subjects of investigation [5,6,7,8,9,10,11,12,13,14,15]. Both weed interference and herbicide treatment stimulate the synthesis of antioxidant molecules in plants in response to stress [10,11,15,16]. Alterations in enzymatic antioxidants such as superoxide dismutase, catalase, ascorbate peroxidase, and peroxidase have been documented in crops and weeds in response to weed interference [11,13,14,17] and herbicide exposure [1,5,18,19,20] together with the synthesis of ROS and causing lipid peroxidation. Also, antioxidant-related gene expression and protein synthesis appear to be responsive to those stressors [8,9,12,16]. 

The increase in the synthesis of several antioxidant defense compounds by crops in response to weed interference could result in crop energy drains and yield losses. Weeds have a high genetic variation that allows them to survive under different stress conditions [21]. In this way, the evolution of herbicide resistance in weeds is a threat to sustainable agriculture, and their mechanisms have been widely studied. In this review, we focus on ROS production in crops under weed interference and in both crops and weeds after herbicide exposure, as well as antioxidant defense systems against ROS. Also, we discuss molecular approaches that may be used to understand responses to ROS better and increase plant adaptation.

## 2. The Basis of ROS Production and Plant Mechanisms of Defense 

**Figure 1:**
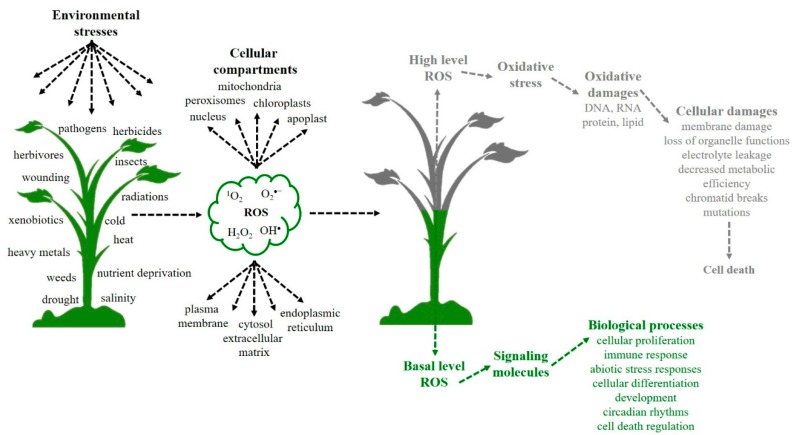
Environmental stressors that can lead to increased generation of reactive oxygen species (ROS) in different cell compartments and the biological consequences. Environmental stressors result in ROS production in different cellular compartments. High-level ROS production results in oxidative stress, oxidative and cellular damages, and even cell death. The basal level of ROS production serves as a signaling molecule and may be involved in various important biological processes.

ROS such as singlet oxygen (^1^O_2_), superoxide radical (O_2_^•−^), hydrogen peroxide (H_2_O_2_), and hydroxyl radical (OH^•^) are partially reduced or excited forms of atmospheric oxygen and considered to be toxic molecules [3]. These molecules have different levels of reactivity, sites of production, and the potential to cross biological membranes [3]. ROS are mainly formed in organelles with high electron flux such as chloroplasts, mitochondria, and peroxisomes [3,4], but also in plant cells in the plasma membrane, cell wall, cytosol, apoplast, endoplasmic reticulum, nucleus, and extracellular matrix (Figure 1) [3,4,22].

In plants, ROS at a basal cellular level is important for signaling molecules capable of regulating diverse metabolic pathways [3,4,23]. Also, these molecules work by activating and controlling gene expression in response to stress [24] and are involved in critical plant biological processes like photosynthesis [2,3]. Thus, the basal level of ROS in cells is essential for plant life [3] and cellular processes. However, high concentrations of ROS can be fatal [2,4,22,25].

The stress generated by biotic and abiotic factors generally induces the overproduction of ROS [2,26]. In response to ROS overproduction, the plant’ defense system acts to scavenge toxic molecules and restore cellular homeostasis. However, when ROS overproduction exceeds the capacity of the antioxidant scavenging system, ROS accumulation occurs [3]. The over-accumulated ROS in plant cells results in lipidic peroxidation (LPO) and membrane damage [2]. LPO is a complex process, which is initiated by hydrogen abstraction or the addition of an oxygen radical. This reaction results in oxidative damage of polyunsaturated fatty acids (PUFA). The initiation step involves transition metal complexes such as Fe and Cu. The O_2_^•−^ and H_2_O_2_ can initiate the reactions. However, OH^•^ is sufficiently reactive and can initiate LPO by the abstraction of a hydrogen atom, in an unsaturated fatty acyl chain of a PUFA residue. In aerobic conditions, fatty acids can be oxygenated to give rise to a lipid peroxyl group (ROO^•^), which can then be propagated via a peroxidation chain reaction by abstracting a hydrogen atom from adjacent PUFA side chains. The resulting lipid hydroperoxide can decompose into several reactive products such as aldehydes (malonyl dialdehyde), alkanes, lipid alkoxyl radicals, lipid epoxides, and alcohols [2]. Therefore, LPO is considered as the most destructive process known to occur in living organisms altering membrane fluidity and permeability, damaging membrane proteins, inactivating receptors as well as other enzymes and ion channels [2,4,27]. After LPO, there can be damage to nucleic acids including chromatid breaks and other mutations, loss of organelle function, reduction in metabolic efficiency, electrolyte leakage, and subsequently, cell death [2,4] (Figure 1). If plants can survive to LPO, a decrease in crop biomass yield is almost always certain [2,3,4,25]. 

**Figure 2:**
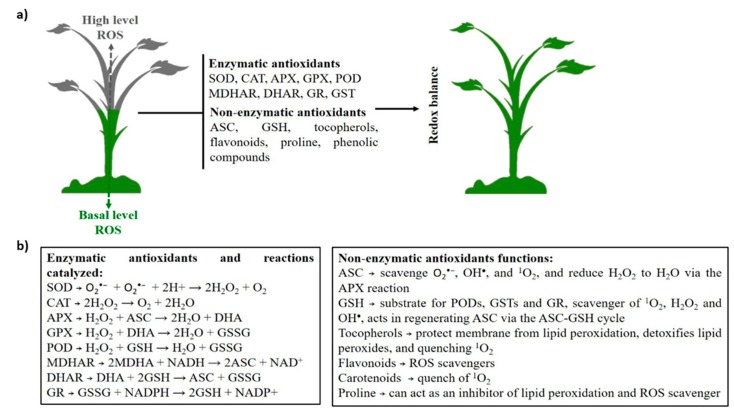
Key enzymatic and non-enzymatic antioxidants and the reactions catalyzed. (**a**) When ROS is overproduced, the enzymatic and non-enzymatic antioxidants act to scavenge the toxic molecules and restore the redox balance. (**b**) Reactions catalyzed by enzymatic antioxidants as well as the functions of the non-enzymatic antioxidants are shown.

The ROS scavenging antioxidant-defense machinery includes enzymatic and non-enzymatic components which serve to balance the production and detoxification of ROS (Figure 2) [2].

Enzymatic antioxidants include superoxide dismutase (SOD), catalase (CAT), ascorbate peroxidase (APX), glutathione peroxidase (GPX), peroxidase (POD), peroxiredoxin (PRX), monodehydroascorbate reductase (MDHAR), dehydroascorbate reductase (DHAR), glutathione reductase (GR), and glutathione-S-transferase (GST) [2,4,22]. SODs are enzymes that catalyze the dismutation of O_2_^•−^ to H_2_O_2_. Thus, SODs enzymes are at the frontline in the defense against ROS. CAT enzymes catalyze the dismutation of two H_2_O_2_ molecules to water and O_2_. These enzymes are localized mainly in peroxisomes. APXs play a key role catalyzing the conversion of H_2_O_2_ into H_2_O using the ascorbate as a specific electron donor. APXs are distributed in chloroplasts, mitochondria, peroxisomes, and the cytosol, coding different isoforms. GPXs catalyze the reduction of H_2_O_2_ or organic hydroperoxides to H_2_O. GPXs are nonheme thiol peroxidases and in plants are localized to mitochondria, chloroplasts, and cytosol. PODs are involved in H_2_O_2_ detoxification. Also, PODs can perform a second cyclic reaction, the hydroxylic reaction, this is distinct from the peroxidative reaction. Further, these enzymes are involved in the biosynthesis of lignin and defense against biotic stresses by consuming H_2_O_2_. MDHARs catalyze the regeneration of ascorbic acid (ASC) from the monodehydroascorbate radical using NAD(P)H as an electron donor. Thus, MDHARs play an essential role in the antioxidant system to maintain the ascorbate pool. DHARs are thiol enzymes that maintain the ascorbate in reduced form. DHARs catalyze the reduction of dehydroascorbate to ascorbate using glutathione (GSH) as a reducing substrate. GR enzymes catalyze the reduction of oxidized glutathione (GSSG) to reduced GSH. GRs are a NAD(P)H-dependent enzyme that protects cells against oxidative damage [2,4,25,26,27,28]. Also, GSTs are isozymes known to protect cells against chemical-induced toxicity. These enzymes catalyze the conjugation of GSH to a variety of electrophilic and hydrophobic substrates [29]. Accordingly, the action of the cellular antioxidant machinery is essential to control excess ROS to protect plant cells from oxidative damage and to restore the redox homeostasis.

Non-enzymatic antioxidants include ASC, glutathione, tocopherol, flavonoids, proline, and phenolic compounds [2,4]. It is important to highlight the role played by non-enzymatic compounds, that are substances found in all cellular compartments and may act directly in the detoxification of ROS and radicals or reduce substrates for antioxidant enzymes [2,3]. ASC is the most abundant, powerful, and water-soluble antioxidant which acts in all tissues, serving as a scavenger for O_2_^•−^, OH^•^, and ^1^O_2_, and can reduce H_2_O_2_ to H_2_O via the APX reaction. Glutathione is considered a powerful intracellular antioxidant localized in all cell compartments [2]. It is a potential scavenger of ^1^O_2_, H_2_O_2_, and OH^•^. GSH acts in regenerating ASC via the ASC-GSH cycle; these have redox potential to interact with numerous components and pathways [2,4,26]. Also, carotenoids serve to quench ^1^O_2_ and flavonoids as ROS scavengers [2]. Tocopherols serve to protect the membrane from lipid peroxidation, detoxify lipid peroxides, and quench ^1^O_2_ [2]. Proline is an osmoprotectant that has been considered to be a powerful antioxidant [4]. Proline can act as an inhibitor of lipid peroxidation and ROS scavenger [2,4]. Phenolic compounds play roles as antioxidants through their ability to donate electrons or hydrogen atoms to ROS [2,4]. Thus, the widespread biochemical activity of non-enzymatic antioxidants can prevent, reduce, or eliminate ROS damage in different plant tissues and cell compartments. 

## 3. Modulation of Enzymes and Antioxidant Genes Induced by Weed Interference and Herbicide Exposure

Plants are immobile organisms that are adapted to cope with suites of environmental stresses present in their native environments. Crops retain some adaptations to deal with stress, but cultivation and crops are largely artificial constructs. One prominent stress in crops comes from weed interference and then also the subsequent application of herbicides to control weeds in modern agriculture. Certainly, these stresses are not completely understood, but play critical roles in crop management, crop development, and reproduction, and often lead to yield loss and reduced farmer profits [26].

### 3.1. Crop and Weed Interference

Weed interference is the most important biotic stress in crop production [30,31]. The level of interference varies with the crop and weed species, growing season, plant spatial distribution and density, the period of coexistence, edaphoclimatic conditions, and potential allelopathy [30,31,32]. Competition is the most common type of interference exerted from weeds on crops, which is established since at least one environmental resource is limited [31]. Competition can occur among plants of different species (interspecific) and the same species (intraspecific—e.g., high crop density). When the competition is established, the species with higher competitive capacity and ability get an advantage to access the environmental resources and grow faster than others, resulting in yield losses to those less competitive [30,32].

Studies have demonstrated alterations in ROS production in crops as a response to weed interference, as well as changes in enzymatic activity and gene expression of antioxidant components [7,11,13,14,33]. Current research is needed to tie weed interference with crop oxidative stress more closely. Especially important is quantifying the dynamic expression of antioxidant genes as well as changes in enzyme activity and compounds as a means of coping with ROS. Finally, it is important to assess the energetics of crops as they cope with oxidative stress and the effects on yield. 

Despite documented alterations in oxidative stress and antioxidant systems, the literature remains divided on the response of crops to weed interference. In soybean and bean, Piasecki et al. [13,14] showed that H_2_O_2_ levels, APX, and CAT activity decreased with the increase in volunteer corn density, while SOD activity increased commensurate with weed density. Another study documented that the interference of wild poinsettia (*Euphorbia heterophylla*) on soybean did not result in any cellular damage or change in SOD, CAT, and APX enzyme activity in soybean [34]. Darmanti et al. [10] studied soybean under purple nutsedge (*Cyperus rotundus* L.) competition and found reduced activities of SOD, CAT, and APX in soybean. 

On the other hand, studies conducted with soybean and wheat under the interference of Italian ryegrass (*Lolium multiflorum*) resulted in oxidative damage and enhanced of the activity of SOD, CAT, and APX enzyme [11,17]. The interference of ryegrass with maize seedlings increased the H_2_O_2_ level and the antioxidant gene expression of *ZmGST1*, *ZmSOD2*, *ZmAPX2*, and *ZmCAT3* [7]. These same authors suggested that the changes which occurred in response to weed interference resulted in a physiological cost to the crop, which contributes to yield loss. Gal et al. [33] studied the interference of ryegrass on soybean and found an increase in H_2_O_2_ content and LPO with a concomitant reduction in flavonoid content in soybean. Also, the transcript levels of the antioxidant genes *apx*, *cat*, *sod*, and *gpx* increased [33], demonstrating that biochemical and molecular mechanisms were altered in soybean under weed interference. 

After stress signals emanate from weed interferences, crop plants activate their antioxidant defense mechanisms that deal with ROS and restore cellular homeostasis [13,14,33]; these defenses certainly have energetic costs vis-à-vis yield. For example, soybean yield is decreased when volunteer maize is a competitor [13]. Although some studies show the involvement of the crop antioxidant system with weed interference, specific details on how it is initiated are lacking and might be related to light conditions [13] and allelopathic compounds exuded from weeds [35]. A consequence of far-red-enriched (FR-E) light is the generation of ROS [7]. Therefore, it is proposed that the FR-E light reflected from neighboring weeds increases the production of ^1^O_2_ which initiates the formation of H_2_O_2_ via ascorbate and disrupts thiol-modulated chloroplast enzymes. This triggers a physiological event that impacts both photosynthesis and carbon partitioning [36]. Allelochemicals stimulate the production of ROS by blocking the electron-carrying chain: electrons become free and react easily with O_2_ to form superoxide [37]. Thus, triggering ROS production and activation of the antioxidant-mediated defense [35] may result in damage to DNA, proteins and cellular membranes. In maize, allelochemical stress was applied by treatment with walnut husk wash water, which possesses allelopathy and phytotoxic effects. The treatment increased H_2_O_2_ content and changed the activity of CAT, SOD, and APX enzyme in maize. In this way, CAT activity increased by 85% in maize roots after 3 h [38]. Furthermore, 4-day juglone treatment (allelochemical) stimulated the expression of the glutathione transferase (*GstI*) gene in maize seedlings [39]. 

Studies of crops under oxidative stress caused by weed interference generally do not assess the yield components and yield. Thus, the understanding of the effective costs for the crop yield as a response to weed interference is limited with regards to ROS. Multiple stresses, ROS dynamics, and crop responses and defenses need to be better understood in relevant field studies as crops proceed from seed to seed. Modern management practices, including herbicide and other pesticide treatments, should also be factored into the stress–yield equation. 

### 3.2. Herbicide Treatment

Herbicides, by definition, cause abiotic stress on plants. Globally, herbicides are the predominant method for controlling weeds in modern crop production, contributing to protecting the crop yield and economic profit [40]. Despite their inherent selectivity mechanisms that facilitate crop production, herbicides can cause some phytotoxicity to crop plants and cause reductions in leaf area index (LAI), shoot dry weight (SDW), plant height, and alterations in plant metabolism by generating ROS [41]. Most of the perturbations caused by herbicide treatment in plants are related to ROS generation and consequent oxidative stress [41].

Numerous herbicide modes of action (MOA) have been commercialized for application to agricultural fields. The herbicide MOA is the physiological and biochemical process (step-by-step) related to herbicide treatment. Each herbicide MOA has a specific target site (TS) which is referred to as a mechanism of action (MA). The target site herbicide is usually an enzyme/protein which is inhibited by herbicides at the molecular level. After herbicide TS inhibition in susceptible plants, at least a vital biological process is interrupted causing a sequence of secondary effects which ultimately leads to plant death [41]. For example, in susceptible plants, glyphosate MA is the inhibition of the enzyme 5-enolpyruvylshikimate 3-phosphate synthase (EPSPS). On the other hand, glyphosate MOA involves processes which occur after EPSPS inhibition, such as the inhibition of the the shikimic acid pathway and biosynthesis of aromatic amino acids (phenylalanine, tyrosine, and tryptophan), accumulation of shikimic acid and reducing power (NADPH+H), ROS generation, oxidative stress, and susceptible plant death from seven to 15 days after treatment [9,42,43].

The MOA for some herbicides induces the generation of ROS in plants as secondary effects after the specific TS is sufficiently inhibited. In this case, the oxidative stress generated is responsible for an important part of cellular and tissue damage. For example, glyphosate action produces ROS as a secondary consequence of the inhibition of the shikimic acid pathway. After the blockage of the shikimic acid pathway, occurs the reducing power accumulation in the chloroplasts. Also, the lack of tyrosine inhibits the synthesis of plastoquinone, which is an electron acceptor in the photosynthetic electron transport chain in the photosystem II (PSII). The non-regeneration of plastoquinone in the PSII interrupts the electron transport, leading to an energy accumulation [41]. Therefore, both processes, reducing power accumulation and PSII blockage, lead to ROS production, cell damage, and plant death [41].

The summary of the major MA according to the Herbicide Resistance Action Committee (HRAC) and whether their actions lead to ROS production in some action step [41] are presented in Table 1. From 21 known groups of herbicides classified by the mode of action described in Table 1, 15, i.e., 71%, causes ROS overproduction after their target site inhibition. Nine of the 15 MA groups (~60%) kill plants by direct ROS production (C1, C2, C3, D, E, F1, F2, F3, and H), whereas the other six, i.e., ~40%, cause ROS production as a secondary effect (B, G, I, M, O, and P) (Table 1). Only six MA herbicides (~29%) (A, K1, K2, K3, L, and N) are not documented to produce oxidative stress in any phase of their action (Table 1) [41]. Also, the use of herbicide-active ingredients from this last group is lower when compared to A, B, and G groups (ACCase, ALS, and EPSPS, respectively) that are the most used herbicides [44]. 

After the active herbicide reaches and inhibits the TS, a series of stress events are initiated by the signaling of plant defense systems against perturbations. The response time for oxidative stress occurrence and visible plant damage varies with the herbicide mode of action, type of herbicide and formulation, plant species, development stage, and environmental conditions [41]. For example, paraquat (photosynthesis inhibitor—PSI) damage could be observed from 2 hours after treatment under light conditions, and susceptible plants die from 3 to 7 days after. On the other hand, the first visible plant symptoms from glyphosate and plant death for susceptible species may occur around 5 days and 7 to 15 days, respectively (Table 1).

The plant antioxidant defense system must act quickly and efficiently to cope with ROS produced by herbicides, especially those which produce ROS directly (Table 1). However, herbicides which produce ROS as a secondary effect may have a lag time between treatment to creating oxidative stress. Thus, as these herbicides cause a wide range of perturbations, likely the antioxidant system acts as a complement to the resistance process. The complexity of all processes involved in plant defense against herbicide action related to NTSR is still poorly understood.

## 4. Coevolution of Herbicide Resistance and Antioxidant Systems 

Weed evolution has been hastened by human action in the last two to three decades, mainly through the intensive use of herbicides [40,46,47]. According to Heap [48], globally, there are 497 unique cases of weeds resistant to herbicides including 23 of 26 known herbicide’s sites of action. These cases comprise 255 species (148 dicots and 107 monocots), reported in 92 crops in 70 countries. Also, weeds have evolved resistance to around 90% of the known herbicide sites of action. 

Herbicide resistance occurs when a plant biotype survives and reproduces after exposure to a normally lethal dose of herbicide [49]. According to Yuan et al. [49] and Délye [47], in weeds there are two primary mechanisms of herbicide resistance: (1) alterations in the herbicide target-site (target-site resistance—TSR) such as mutation and overexpression; and (2) any other alterations which do not involve the herbicide target-site (non-target site resistance—NTSR), such as herbicide absorption, translocation, metabolism, and compensation or protection [44]. In general, TSR resistance is monogenic, and as herbicides are developed to target specific enzymes or proteins, a simple point mutation could alter the enzyme structure and confer the resistance, making the herbicide treatment ineffective. On the other hand, NTSR is a complex polygenic adaptation to herbicides which involves a multi-step process [44,49]. NTSR is considered the predominant type of resistance and comprises a very complex molecular process which remains to be fully comprehended [43]. This type of resistance is a serious threat for agriculture because it can confer cross-resistance to various modes of action herbicides (multiple herbicide resistance), including those not yet discovered [44,49].

The NTSR can be caused by a plant detoxification process that follows a four-phase: I—detoxification; II—conjugation; III—transport; and IV—degradation [44,47,49]. In the first phase, detoxification involves the oxidation process carried out by P450 monooxygenases or oxidases with various functions. In the second phase, conjugation of xenobiotic occurs by the addition of thiols or sugars, or directly by glutathione S-transferases and glycosyltransferases. The third phase comprises the transport of the conjugated molecule into the vacuole or extracellular space by ABC transporters, which are the most known group of transporters. Finally, the fourth phase involves degradation of the conjugated molecule [44,47,49].

Antioxidant systems and protection against oxidative damage caused by herbicide action plays an important role in NTSR [9,44,50,51,52]. This system could act to directly scavenge ROS or complement other herbicide resistance mechanisms in a complex set of coordinated processes [9]. In this way, as ~70% of MA herbicides involve ROS production and oxidative stress (Table 1), this type of resistance can cause a multi-herbicide resistance, representing a threat to sustainable agriculture and worries scientists, herbicides developers, and farmers. 

Research has shown modulations in the scavenging activity of ROS within the antioxidant defense machinery in plants exposed to the herbicide. In general, the antioxidant enzyme activities, ROS levels, and LPO are elevated. However, in some cases, high herbicide concentrations decrease the antioxidant enzyme activities. Table 2 shows the percentage values to variations in antioxidants enzyme, ROS, and LPO (Table 2). 

In multiple herbicide-resistant (MHR) wild oats (*Avena fatua*), redox-related enzymes with elevated expression were identified, suggesting that these plants exhibit a high capacity for redox maintenance since these plants are resistant to 11 herbicides from five different MA [12]. Also, the authors [12] described that the transcriptional regulation of MHR was similar to those presented by phenotypes tolerant to abiotic stress. For goosegrass (*Eleusine indica* L. (Gaertn)), the analysis of the differentially expressed genes revealed that most ROS pathway genes were up-regulated in resistant and susceptible biotypes after the application of paraquat [8]. In wheat, prometryne and symetrine-induced oxidative stress and increased transcript abundance of *Cu/Zn-SOD*, *GR*, *APX*, and *GST* genes in leaves and roots [5,6,56]. The *Cu/Zn-SOD* expression in leaves increased 2.6-fold with 12 mg kg^−1^ prometryne [5], and *Cu/Zn-SOD* and *GST* genes in leaves with 8 mg kg^−1^ prometryne increased 7.6 and 4.4-fold, respectively [6]. Also, the effects of simetryne on transcript abundance of *Cu/Zn-SOD*, *GR*, *APX,* and *GST* genes showed an increase of 2.1, 1.3, 1.6, and 1.4-fold, respectively [56]. Furthermore, in rice, atrazine treatment resulted in *OsGST3*, *OsGST4*, *OsAPX2*, *OsAPX3*, *OsGR1*, and *OsGR3* genes up-regulation [18]. These results indicate that antioxidants gene can be highly regulated by herbicides at the molecular level in plants. Thus, crops are capable of activating antioxidants mechanisms to alleviate herbicide-induced stress. In rice, atrazine treatment resulted in *OsGST3*, *OsGST4*, *OsAPX2*, *OsAPX3*, *OsGR1*, and *OsGR3* genes up-regulation [18]. Maroli et al. [9] demonstrated the potential role of antioxidant systems in glyphosate resistance in Palmer amaranth (*Amaranthus palmeri*) using metabolic profile and enzyme analyses. Harre et al. [15] documented the involvement of antioxidant enzymes in the glyphosate resistance in *Ambrosia trifida*.

In rice, the over-expression of the *OsGSTL2* gene improved glyphosate and chlorsulfuron tolerance [60]. Transgenic plants contained higher levels of GST activities, 2.1, 1.8 and 2.4-fold, which were enough to provide tolerance to herbicides. Further, in transgenic plants, the GPX activity was higher, 1.6, 1.5 and 1.7-fold of that detected in non-transformed plants; the GPX enzyme, therefore, can help degrade superoxide levels [60]. Transgenic potato overexpressing *Arabidopsis* cytosolic *AtDHAR1* gene exhibited increased DHAR activity up to 4.5 times, as well as higher tolerance to methyl viologen herbicide [61]. These plants, when subjected to herbicide, exhibited less ion leakage, greater chlorophyll contents, less accumulation of H_2_O_2_, and less severe visual injury symptoms [61], demonstrating the power of the antioxidant system.

## 5. Transcriptomic/Proteomic Approaches Helping to Clarify the Antioxidant Response to Herbicides in Plants 

Transcriptomics and proteomic approaches have helped to clarify the genetic regulation in response to herbicide treatment [9,44,47]. Transcriptomic and proteomic approaches permit the characterization of the expression levels of genes or gene families and proteins for important biochemical pathways in response to herbicide treatment. Thus, in these studies the expression levels of metabolic pathways may change during the organism life cycle and tissue, and according to the environmental conditions. 

RNA-sequencing is the preferred method these days to identify transcripts that are differentially expressed in a genome in target tissues under environmental treatments. Differential expression of genes related to antioxidant pathways following herbicide treatment of weeds is important to characterize in species with evolved herbicide resistance (Table 3).

These studies have shown that several antioxidants genes have altered expression in weeds after herbicide treatment. The data shows how antioxidant defense mechanisms were activated in response to herbicide, thus working to restore redox homeostasis. Considering all aspects indicated above, the data suggests that these weedy species exhibit a high capacity for redox maintenance. The synergistic transcriptome/proteomic combination provides a complete representation of the plant phenotype [12]. Accordingly, the proteomic analyses of the rice plants treated with glyphosate herbicide demonstrated that antioxidant proteins APX, GST, PRX, and SOD were accumulated. Further, the transcript levels of the genes GST, PRX, and APX increased [16], suggesting that the herbicide generated oxidative stress.

## 6. Concluding Remarks

ROS overproduction occurs in plants exposed to environmental stresses. ROS are potential toxic molecules that are damaging to cell components and can lead to cell death. The role of antioxidant response systems is to modulate ROS during periods of normal growth or in response to stress to regulate cellular redox homeostasis.

Weed interference is consistently a biotic stressor in crops; both weed interference and herbicide treatment can lead to ROS accumulation in crops as indicated by increased O_2_^•−^ and H_2_O_2_ levels. Various crop species and genotypes vary in these responses in space and time, as well as by degree of interference and herbicide treatment. Lipid peroxidation responses from ROS overproduction are a pernicious effect that commonly causes reduced crop biomass and yield.

Herbicides remain as the primary tool for implementing weed management in the major agronomic crops of the world. Of the 21 herbicide mechanisms of action we described, nearly 43% cause direct ROS production and an additional 29% are indirectly involved in increased ROS in plants. Weeds may have increased the propensity to evolve antioxidant defenses compared with crops. Weedy biotypes with herbicide resistance, especially NTSR to multiple herbicides, appear to have enhanced mechanisms to deal with ROS. It is critical to better understand the interplay of evolved antioxidant responses at the molecular level in weeds. 

To this end, transcriptomic and proteomic approaches are beginning to illuminate which antioxidant defense mechanisms are activated in response to ROS and their roles to maintain redox homeostasis. Reverse genetics studies in crops that alter antioxidant enzyme profiles for stress tolerance are an important approach for crop improvement and to improve our understanding of basic cellular mechanisms. Moreover, the application of exogenous protectants such as plant nutrients, antioxidants, osmolytes, phytohormones, signaling molecules, and others have been employed and may contribute to mitigating the toxic effects of a high ROS level through increasing the antioxidant defenses in crops.

## Figures and Tables

**Table 1:** Description of the herbicide mechanisms of action (MA) and their effects on treated plants related to the production of reactive oxygen species.

HRAC Group ^a^	Herbicide Mechanism of Action (MA)	Biological Process Committed	Herbicide Chemical Family	Herbicide Active Ingredient ^b^	ROS Production ^c^
A	Inhibition of acetyl-CoA carboxylase (ACCase)	Fatty acid biosynthesis	Aryloxyphenoxy-propionate “FOPS”, Cyclohexanedione “DIMs”, Phenylpyrazoline “DEN”	benzoylprop-ethyl, diclofop-methyl, haloxyfop-methyl, cyhalofop, clethodim, setoxydim, pinoxaden	No
B	Inhibition of acetohydroxyacid synthase (AHAS, ALS)	Amino acid biosynthesis (Leu, Ile, Val)	Sulfonylurea, Imidazolinone, Triazolopyrimidine, Pyrimidinyl(thio)benzoate	metsulfuron-methyl, chlorimuron-ethyl, nicosulfuron, imazapyr, imazethapyr, flumetsulam, cloransulam-methyl, diclosulam, flucarbazone-sodium, pyritiobac	Yes
C1, C2, C3	Inhibition of photosystem II protein D1 (psbA)	Photosynthesis (electron transfer)	Triazine, Triazinone, Triazolinone, Uracil, Pyridazinone, Phenyl-carbamate, Urea, Amide, Nitrile, Benzothiadiazinone, Phenyl-pyridazine	ametryne, atrazine, simazine, hexazinone, metribuzin, amicarbazone, bromacil, pyrazon, desmedipham, chlorotoluron, diuron, linuron, propanil, bromoxynil, ioxynil, bentazon, pyridate	Yes
D	Diversion of the electrons transferred by the photosystem I ferredoxin (Fd)	Photosynthesis (electron transfer)	Bipyridylium	diquat, paraquat	Yes
E	Inhibition of protoporphyrinogen oxidase (PPO)	Photosynthesis (heme biosyn- thesis for chlorophyll)	Diphenylether, Phenylpyrazole, N-phenylphthalimide, Thiadiazole, Oxadiazole, Triazolinone, Oxazolidinedione, Pyrimidindione	acifluorfen-Na, fomesafen, lactofen, oxyfluorfen, pyraflufen-ethyl, flumioxazin, fluthiacet-methyl, oxadiazon, azafenidin, pentoxazone, butafenacil, flufenpyr-ethyl	Yes
F1, F2, F3	Inhibition of phytoene desaturase (PDS) or 4-hydroxyphenylpyruvate dioxygenase (4-HPPD) or of an unknown protein	Photosynthesis (carotenoid biosynthesis)	Pyridazinone, Pyridinecarboxamide, Triketone, Isoxazole, Pyrazole, Triazole, Isoxazolidinone, Urea, Diphenylether	norflurazon, diflufenican, fluridone, mesotrione, isoxaflutole, pyrazoxyfen, amitrole, clomazone, fluometuron, aclonifen	Yes
G	Inhibition of 5-enolpyruvylshikimate-3-phosphate synthase (EPSPS)	Amino acid biosynthesis (Phe, Trp, Tyr)	Glycine	glyphosate	Yes
H	Inhibition of glutamine synthase	Amino acid biosynthesis (Gln)	Phosphinic acid	glufosinate-ammonium	Yes
I	Inhibition of dihydropteroate synthase	Tetrahydrofolate biosynthesis	Carbamate	asulam	Yes
K1, K2	Enhancement of tubulin depolymerization	Microtubule polymerization	Dinitroaniline, Phosphoroamidate, Pyridine, Benzamide, Benzoic acid, Carbamate	oryzalin, pendimethalin, trifluralin, amiprophos-methyl, dithiopyr, propyzamide, DCPA, carbetamide	No
K3	Inhibition of fatty acid synthase (FAS)	Fatty acid biosynthesis	Chloroacetamide, Acetamide, Oxyacetamide, Tetrazolinone	acetochlor, alachlor, metolachlor, napropamide, flufenacet, fentrazamide, anilofos	No
L	Inhibition of cellulose-synthase	Cell wall biosynthesis	Nitrile, Benzamide, Triazolocarboxamide, Quinoline carboxylic acid	dichlobenil, isoxaben, flupoxam, quinclorac	No
M	Uncoupling of oxidative phosphorylation	ATP biosynthesis	Dinitrophenol	dinoseb, dinoterb	Yes
N	Inhibition of fatty acid elongase	Fatty acid biosynthesis	Thiocarbamate, Phosphorodithioate, Benzofuran, Chloro-Carbonic-acid	butylate, cycloate, EPTC, bensulide, ethofumesate, TCA, dalapon	No
O	Stimulation of transport inhibitor response protein 1 (TIR1)	Regulation of auxin-responsive genes	Phenoxy-carboxylic-acid, Benzoic acid, Pyridine carboxylic acid, Quinoline carboxylic acid	2,4-D, MCPA, dicamba, clopyralid, fluroxypyr, picloram, triclopyr, quinclorac, quinmerac, benazolin-ethyl	Yes
P	Inhibition of auxin transport	Long-range hormone signaling	Phthalamate, Semicarbazone	naptalam, diflufenzopyr-Na	Yes

^a^ The 21 known groups of herbicides classified by mechanims of action (MA) according to the global Herbicide Resistance Action Committee (HRAC; http://www.hracglobal.com, accessed on: 28 January 2019. Also, there are other MAs, but they are not completely understood as the biochemical processes were not described. Please see the HRAC website to check all MA. ^b^ Not all active herbicide ingredients are presented, please see the HRAC website to check all of them. ^c^ Indicates the ROS production in some phase of the herbicide action [41,45].

**Table 2:** Alterations in antioxidant enzyme activities, ROS level, and lipid peroxidation in different species after herbicide treatment.

Species	Herbicide Concentration	Time	Tissue	* Antioxidants Enzyme	* ROS Level	* Lipid Peroxidation	References
*Triticum aestivum* L.	Chlorotoluron 0, 5, 10, 15, 20 25 mg kg^−1^	10 days	Roots Leaves	CAT ↑ 5 mg kg^−1^ (80%); ↑ 10 mg kg^−1^ (35%); ↑ 15 mg kg^−1^ (5%); ↓ 20 mg kg^−1^ (11%); ↓ 25 mg kg^−1^ (23%); SOD ↑ 5 mg kg^−1^ (100%); ↑ 10 mg kg^−1^ (200%); ↑ 15 mg kg^−1^ (300%); ↑ 20 mg kg^−1^ (430%); ↑ 25 mg kg^−1^ (500%); APX ↑ 5 mg kg^−1^ (160%); ↑ 10 mg kg^−1^ (260%); ↑ 15 mg kg^−1^ (80%); ↑ 20 mg kg^−1^ (70%); ↑ 25 mg kg^−1^ (40%); POD ↑ 5 mg kg^−1^ (88%); ↑ 10 mg kg^−1^ (233%); ↑ 15 mg kg^−1^ (210%); ↑ 20 mg kg^−1^ (188%); ↑ 25 mg kg^−1^ (133%); CAT ↓ 5 mg kg^−1^ (17%); ↓ 10 mg kg^−1^ (23%); ↓ 15 mg kg^−1^ (35%); ↓ 20 mg kg^−1^ (41%); ↓ 25 mg kg^−1^ (47%); SOD ↑ 5 mg kg^−1^ (4%); ↑ 10 mg kg^−1^ (60%); ↑ 15 mg kg^−1^ (180%); ■ 20 mg kg^−1^ (0%); ↓ 25 mg kg^−1^ (4%); APX ↑ 5 mg kg^−1^ (100%); ↑ 10 mg kg^−1^ (300%); ↑ 15 mg kg^−1^ (75%); ↑ 20 mg kg^−1^ (50%); ↑ 25 mg kg^−1^ (25%); POD ■	nd ^†^H_2_O_2_ ↑ ^†^O_2_^•−^ ↑	■ 5 mg kg^−1^ (0%) ↑ 10 mg kg^−1^ (50%) ↑ 15 mg kg^−1^ (40%) ↑ 20 mg kg^−1^ (35%) ■ 25 mg kg^−1^ (0%) ↑ 5 mg kg^−1^ (125%) ↑ 10 mg kg^−1^ (225%) ↑ 15 mg kg^−1^ (150%) ↑ 20 mg kg^−1^ (50%) ↑ 25 mg kg^−1^ (25%)	[19]
*Triticum aestivum* L.	Prometryne 0, 4, 8, 12, 16, 20, 24 mg kg^−1^	10 days	Roots Leaves	CAT ↓ 4 mg kg^−1^ (20%); ↓ 8 mg kg^−1^ (24%); ↓ 12 mg kg^−1^ (37%); ↓ 16 mg kg^−1^ (42%); ↓ 20 mg kg^−1^ (48%); ↓ 24 mg kg^−1^ (55%); SOD ↑ 4 mg kg^−1^ (14%); ↑ 8 mg kg^−1^ (52%); ↑ 12 mg kg^−1^ (45%); ■ 16 mg kg^−1^ (0%); ■ 20 mg kg^−1^ (0%); ↓ 24 mg kg^−1^ (37%); APX ■ 4 mg kg^−1^ (0%); ↑ 8 mg kg^−1^ (23%); ↑ 12 mg kg^−1^ (44%); ↑ 16 mg kg^−1^ (16%); ■ 20 mg kg^−1^ (0%); ↓ 24 mg kg^−1^ (25%); POD ↑ 4 mg kg^−1^ (58%); ↑ 8 mg kg^−1^ (76%); ↑ 12 mg kg^−1^ (66%); ↑ 16 mg kg^−1^ (58%); ↑ 20 mg kg^−1^ (23%); ■ 24 mg kg^−1^ (0%); GST ↑ 4 mg kg^−1^ (50%); ↑ 8 mg kg^−1^ (64%); ↑ 12 mg kg^−1^ (57%); ↑ 16 mg kg^−1^ (42%); ↑ 20 mg kg^−1^ (21%); ■ 24 mg kg^−1^ (0%); CAT ↑ 4 mg kg^−1^ (21%); ↑ 8 mg kg^−1^ (50%); ↑ 12 mg kg^−1^ (42%); ↑ 16 mg kg^−1^ (21%); ■ 20 mg kg^−1^ (0%); ↓ 24 mg kg^−1^ (30%); SOD ■ 4 mg kg^−1^ (0%); ↑ 8 mg kg^−1^ (57%); ↑ 12 mg kg^−1^ (68%); ↑ 16 mg kg^−1^ (47%); ↑ 20 mg kg^−1^ (0%); ■ 24 mg kg^−1^ (0%); APX ↑ 4 mg kg^−1^ (16%); ↑ 8 mg kg^−1^ (36%); ↑ 12 mg kg^−1^ (56%); ↑ 16 mg kg^−1^ (66%); ↑ 20 mg kg^−1^ (43%); ↑ 24 mg kg^−1^ (26%); POD ■ 4 mg kg^−1^ (0%); ↑ 8 mg kg^−1^ (37%); ↑ 12 mg kg^−1^ (43%); ↑ 16 mg kg^−1^ (18%); ■ 20 mg kg^−1^ (0%); ■ 24 mg kg^−1^ (0%); GST ■ 4 mg kg^−1^ (0%); ↑ 8 mg kg^−1^ (100%); ↑ 12 mg kg^−1^ (116%); ↑ 16 mg kg^−1^ (50%); ↑ 20 mg kg^−1^ (16%); ↓ 24 mg kg^−1^ (33%);	nd	↑ 4 mg kg^−1^ (250%) ↑ 8 mg kg^−1^ (450%) ↑ 12 mg kg^−1^ (400%) ↑ 16 mg kg^−1^ (325%) ↑ 20 mg kg^−1^ (275%) ↑ 24 mg kg^−1^ (250%) ↑ 4 mg kg^−1^ (140%) ↑ 8 mg kg^−1^ (260%) ↑ 12 mg kg^−1^ (200%) ↑ 16 mg kg^−1^ (140%) ↑ 20 mg kg^−1^ (135%) ↑ 24 mg kg^−1^ (135%)	[5]
*Brassica napus* L. *Brassica rapa* L.	ZJ0273 0, 100, 500, 1000 mg L^−1^	7 days 14 days 28 days 7 days 14 days 28 days	Leaves	SOD ↓ 100 mg L^−1^ (5%); ↓ 500 mg L^−1^ (22%); ↓ 1000 mg L^−1^ (38%); SOD ↓ 100 mg L^−1^ (3%); ↓ 500 mg L^−1^ (17%); ↓ 1000 mg L^−1^ (28%); SOD ↓ 100 mg L^−1^ (8%); ↓ 500 mg L^−1^ (21%); ↓ 1000 mg L^−1^ (33%); POD ↓ 100 mg L^−1^ (5%); ↓ 500 mg L^−1^ (42%); ↓ 1000 mg L^−1^ (55%); POD ↓ 100 mg L^−1^ (6%); ↓ 500 mg L^−1^ (33%); ↓ 1000 mg L^−1^ (47%); POD ↓ 100 mg L^−1^ (1%); ↓ 500 mg L^−1^ (3%); ↓ 1000 mg L^−1^ (29%); SOD ↓ 100 mg L^−1^ (10%); ↓ 500 mg L^−1^ (22%); ↓ 1000 mg L^−1^ (34%); SOD ↓ 100 mg L^−1^ (2%); ↓ 500 mg L^−1^ (17%); ↓ 1000 mg L^−1^ (29%); SOD ↓ 100 mg L^−1^ (1%); ↓ 500 mg L^−1^ (8%); ↓ 1000 mg L^−1^ (15%); POD ↓ 100 mg L^−1^ (9%); ࢑ 500 mg L^−1^ (36%); ↓ 1000 mg L^−1^ (49%); POD ↓ 100 mg L^−1^ (5%); ↓ 500 mg L^−1^ (21%); ↓ 1000 mg L^−1^ (45%); POD ↓ 100 mg L^−1^ (1%); ↓ 500 mg L^−1^ (8%); ↓ 1000 mg L^−1^ (24%);	nd	(7) ↑ 100 mg L^−1^ (9%) ↑ 500 mg L^−1^ (53%) ↑ 1000 mg L^−1^ (58%) (14) ■ 100 mg L^−1^ (0%) ↑ 500 mg L^−1^ (32%) ↑ 1000 mg L^−1^ (44%) (28) ↓ 100 mg L^−1^ (9%) ■ 500 mg L^−1^ (0%) ↑ 1000 mg L^−1^ (1%) (7) ↑ 100 mg L^−1^ (32%) ↑500 mg L^−1^ (86%) ↑ 1000 mg L^−1^ (101%) (14) ↑ 100 mg L^−1^ (25%) ↑ 500 mg L^−1^ (45%) ↑ 1000 mg L^−1^ (63%) (28) ■ 100 mg L^−1^ (0%) ↑ 500 mg L^−1^ (11%) ↑ 1000 mg L^−1^ (19%)	[53]
*Oryza sativa* L.	Fluroxypyr 0, 0.05, 0.1, 0.2, 0.4, 0.8 mg L^−1^	6 days	Roots Leaves	CAT ■ SOD ↑ 0.05 mg L^−1^ (18%); ↑ 0.1 mg L^−1^ (20%); ↑ 0.2 mg L^−1^ (32%); ↑ 0.4 mg L^−1^ (22%); ↑ 0.8 mg L^−1^ (13%); APX ↑ 0.05 mg L^−1^ (10%); ↑ 0.1 mg L^−1^ (15%); ↑ 0.2 mg L^−1^ (10%); ■ 0.4 mg L^−1^ (0%); ↓ 0.8 mg L^−1^ (10%); POD ↑ 0.05 mg L^−1^ (50%); ↑ 0.1 mg L^−1^ (57%); ↑ 0.2 mg L^−1^ (90%); ↑ 0.4 mg L^−1^ (93%); ↑ 0.8 mg L^−1^ (110%); CAT ■ 0.05 mg L^−1^ (0%); ↑ 0.1 mg L^−1^ (15%); ■ 0.2 mg L^−1^ (0%); ↓ 0.4 mg L^−1^ (10%); ↓ 0.8 mg L^−1^ (30%); SOD ↑ 0.05 mg L^−1^ (20%); ↑ 0.1 mg L^−1^ (35%); ↑ 0.2 mg L^−1^ (40%); ↑ 0.4 mg L^−1^ (35%); ↑ 0.8 mg L^−1^ (30%); APX ■ POD ■ 0.05 mg L^−1^ (0%); ■ 0.1 mg L^−1^ (0%); ■ 0.2 mg L^−1^ (0%); ↑ 0.4 mg L^−1^ (45%); ↑ 0.8 mg L^−1^ (55%);	^†^H_2_O_2_ ↑ ^†^O_2_^•−^ ↑	↑ 0.05 mg L^−1^ (17%) ↑ 0.1 mg L^−1^ (25%) ↑ 0.2 mg L^−1^ (45%) ↑ 0.4 mg L^−1^ (40%) ↑ 0.8 mg L^−1^ (17%) ■ 0.05 mg L^−1^ (0%) ↑ 0.1 mg L^−1^ (10%) ↑ 0.2 mg L^−1^ (13%) ↑ 0.4 mg L^−1^ (22%) ↑ 0.8 mg L^−1^ (9%)	[54]
*Zea mays* L.	Clethodim 0, 50, 100, 200, 500, 1000 ppm	21 days	Leaves	CAT ↓ 50 ppm (57%); ↓ 100 ppm (47%); ↓ 200 ppm (23%); ↓ 500 ppm (15%); ↓ 1000 ppm (9%); SOD ↓ 50 ppm (13%); ↓ 100 ppm (25%); ↓ 200 ppm (35%); ↓ 500 ppm (35%); ↓ 1000 ppm (32%); APX ↑ 50 ppm (90%); ↑ 100 ppm (175%); ↑ 200 ppm (82%); ↑ 500 ppm (75%); ↑ 1000 ppm (17%); POD ↑ 50 ppm (92%); ↑ 100 ppm (77%); ↑ 200 ppm (180%); ↑ 500 ppm (123%); ↑ 1000 ppm (190%);	H_2_O_2_↑ 50 ppm (1%) ↑ 100 ppm (23%) ↑ 200 ppm (36%) ↑ 500 ppm (50%) ↑ 1000 ppm (63%)	↑ 50 ppm (45%) ↓ 100 ppm (7%) ↑ 200 ppm (67%) ↑ 500 ppm (120%) ↑ 1000 ppm (182%)	[55]
*Oryza sativa* L.	Atrazine 0, 0.05, 0.1, 0.2, 0.4, 0.8 mg L^−1^	6 days	Roots Leaves	CAT ■ 0.05 mg L^−1^ (0%); ↑ 0.1 mg L^−1^ (25%); ↑ 0.2 mg L^−1^ (25%); ↑ 0.4 mg L^−1^ (25%); ■ 0.8 mg L^−1^ (0%); SOD ■ 0.05 mg L^−1^ (0%); ↑ 0.1 mg L^−1^ (60%); ↑ 0.2 mg L^−1^ (75%); ↑ 0.4 mg L^−1^ (150%); ↑ 0.8 mg L^−1^ (95%); APX ■ 0.05 mg L^−1^ (0%); ↑ 0.1 mg L^−1^ (25%); ↑ 0.2 mg L^−1^ (65%); ↑ 0.4 mg L^−1^ (70%); ↑ 0.8 mg L^−1^ (25%); POD ■ 0.05 mg L^−1^ (0%); ■ 0.1 mg L^−1^ (0%); ↑ 0.2 mg L^−1^ (65%); ↑ 0.4 mg L^−1^ (85%); ↑ 0.8 mg L^−1^ (125%); GST ■ 0.05 mg L^−1^ (0%); ■ 0.1 mg L^−1^ (0%); ↓ 0.2 mg L^−1^ (50%); ↓ 0.4 mg L^−1^ (58%); ↓ 0.8 mg L^−1^ (50%); GR ■ 0.05 mg L^−1^ (0%); ↑ 0.1 mg L^−1^ (25%); ↑ 0.2 mg L^−1^ (100%); ↑ 0.4 mg L^−1^ (50%); ↑ 0.8 mg L^−1^ (40%); CAT ↑ 0.05 mg L^−1^ (50%); ↑ 0.1 mg L^−1^ (100%); ↑ 0.2 mg L^−1^ (125%); ↑ 0.4 mg L^−1^ (150%); ↑ 0.8 mg L^−1^ (200%); SOD ■ 0.05 mg L^−1^ (0%); ↑ 0.1 mg L^−1^ (40%); ↑ 0.2 mg L^−1^ (50%); ↑ 0.4 mg L^−1^ (140%); ↑ 0.8 mg L^−1^ (300%); APX ■ 0.05 mg L^−1^ (0%); ■ 0.1 mg L^−1^ (0%); ↑ 0.2 mg L^−1^ (40%); ↑ 0.4 mg L^−1^ (45%); ■ 0.8 mg L^−1^ (0%); POD ■ 0.05 mg L^−1^ (0%); ↑ 0.1 mg L^−1^ (40%); ↑ 0.2 mg L^−1^ (45%); ↑ 0.4 mg L^−1^ (360%); ↑ 0.8 mg L^−1^ (540%); GST ■ 0.05 mg L^−1^ (0%); ■ 0.1 mg L^−1^ (0%); ↑ 0.2 mg L^−1^ (50%); ↑ 0.4 mg L^−1^ (50%); ↑ 0.8 mg L^−1^ (40%); GR ■ 0.05 mg L^−1^ (0%); ↑ 0.1 mg L^−1^ (50%); ↑ 0.2 mg L^−1^ (95%); ↑ 0.4 mg L^−1^ (150%); ↑ 0.8 mg L^−1^ (115%);	nd ^†^H_2_O_2_ ↑ ^†^O_2_^•−^ ↑	■ 0.05 mg L^−1^ (0%) ↑ 0.1 mg L^−1^ (22%) ↑ 0.2 mg L^−1^ (33%) ↑ 0.4 mg L^−1^ (45%) ↑ 0.8 mg L^−1^ (22%) ■ 0.05 mg L^−1^ (0%) ↑ 0.1 mg L^−1^ (25%) ↑ 0.2 mg L^−1^ (25%) ↑ 0.4 mg L^−1^ (37%) ↑ 0.8 mg L^−1^ (25%)	[18]
*Triticum aestivum* L.	Simetryne 0, 0.8, 1.6, 3.2, 4.8, 6.4, 8.0 mg kg^−1^	7 days	Roots Leaves	CAT ↑ 0.8 mg kg^−1^ (43%); ↑ 1.6 mg kg^−1^ (73%); ↑ 3.2 mg kg^−1^ (15%); ↓ 4.8 mg kg^−1^ (30%); ↓ 6.4 mg kg^−1^ (45%); ↓ 8.0 mg kg^−1^ (70%); SOD ↑ 0.8 mg kg^−1^ (25%); ↑ 1.6 mg kg^−1^ (65%); ↑ 3.2 mg kg^−1^ (105%); ↑ 4.8 mg kg^−1^ (60%); ↑ 6.4 mg kg^−1^ (40%); ↑ 8.0 mg kg^−1^ (20%); APX ↑ 0.8 mg kg^−1^ (50%); ↑ 1.6 mg kg^−1^ (90%); ↑ 3.2 mg kg^−1^ (135%); ↑ 4.8 mg kg^−1^ (120%); ↑ 6.4 mg kg^−1^ (65%); ↑ 8.0 mg kg^−1^ (50%); POD ↑ 0.8 mg kg^−1^ (10%); ↑ 1.6 mg kg^−1^ (50%); ↑ 3.2 mg kg^−1^ (100%); ↑ 4.8 mg kg^−1^ (80%); ↑ 6.4 mg kg^−1^ (30%); ■ 8.0 mg kg^−1^ (0%); GST ↑ 0.8 mg kg^−1^ (20%); ↑ 1.6 mg kg^−1^ (25%); ↑ 3.2 mg kg^−1^ (75%); ↑ 4.8 mg kg^−1^ (55%); ↑ 6.4 mg kg^−1^ (30%); ↑ 8.0 mg kg^−1^ (20%); GR ↑ 0.8 mg kg^−1^ (45%); ↑ 1.6 mg kg^−1^ (90%); ↑ 3.2 mg kg^−1^ (170%); ↑ 4.8 mg kg^−1^ (150%); ↑ 6.4 mg kg^−1^ (140%); ↑ 8.0 mg kg^−1^ (95%); CAT ↑ 0.8 mg kg^−1^ (25%); ↑ 1.6 mg kg^−1^ (85%); ↑ 3.2 mg kg^−1^ (150%); ↑ 4.8 mg kg^−1^ (100%); ↑ 6.4 mg kg^−1^ (50%); ↓ 8.0 mg kg^−1^ (25%); SOD ↑ 0.8 mg kg^−1^ (40%); ↑ 1.6 mg kg^−1^ (110%); ↑ 3.2 mg kg^−1^ (195%); ↑ 4.8 mg kg^−1^ (145%); ↑ 6.4 mg kg^−1^ (100%); ↑ 8.0 mg kg^−1^ (50%); APX ↑ 0.8 mg kg^−1^ (20%); ↑ 1.6 mg kg^−1^ (45%); ↑ 3.2 mg kg^−1^ (100%); ↑ 4.8 mg kg^−1^ (90%); ↑ 6.4 mg kg^−1^ (65%); ↑ 8.0 mg kg^−1^ (45%); POD ■ 0.8 mg kg^−1^ (0%); ↑ 1.6 mg kg^−1^ (15%); ↑ 3.2 mg kg^−1^ (35%); ↑ 4.8 mg kg^−1^ (15%); ↑ 6.4 mg kg^−1^ (10%); ■ 8.0 mg kg^−1^ (0%); GST ↑ 0.8 mg kg^−1^ (10%); ↑ 1.6 mg kg^−1^ (25%); ↑ 3.2 mg kg^−1^ (50%); ↑ 4.8 mg kg^−1^ (70%); ↓ 6.4 mg kg^−1^ (25%); ↓ 8.0 mg kg^−1^ (35%); GR ■ 0.8 mg kg^−1^ (0%); ↑ 1.6 mg kg^−1^ (10%); ↑ 3.2 mg kg^−1^ (25%); ↑ 4.8 mg kg^−1^ (10%); ↑ 6.4 mg kg^−1^ (5%); ■ 8.0 mg kg^−1^ (0%);	^†^H_2_O_2_ ↑ ^†^O_2_^•−^ ↑	↑ 0.8 mg kg^−1^ (20%) ↑ 1.6 mg kg^−1^ (20%) ↑ 3.2 mg kg^−1^ (45%) ↑ 4.8 mg kg^−1^ (25%) ↑ 6.4 mg kg^−1^ (20%) ■ 8.0 mg kg^−1^ (0%) ↑ 0.8 mg kg^−1^ (15%) ↑ 1.6 mg kg^−1^ (35%) ↑ 3.2 mg kg^−1^ (45%) ↑ 4.8 mg kg^−1^ (35%) ↑ 6.4 mg kg^−1^ (15%) ↑ 8.0 mg kg^−1^ (10%)	[56]
*Phaseolus vulgaris* L.	Prometryn 0, 10, 100, 500 µM	21 days	Roots Leaves	CAT ↑ 10 µM (35%); ↑ 100 µM (40%); ■ 500 µM (0%); APX ↑ 10 µM (35%); ↑ 100 µM (70%); ↓ 500 µM (22%); GST ↑ 10 µM (8%); ↑ 100 µM (15%); ↓ 500 µM (11%); CAT ↑ 10 µM (30%); ↑ 100 µM (100%); ↓ 500 µM (25%); APX ↑ 10 µM (20%); ↑ 100 µM (42%); ↓ 500 µM (49%); GST ↑ 10 µM (55%); ↑ 100 µM (110%); ↓ 500 µM (18%);	nd nd	nd ■ 10 µM (0%) ↑ 100 µM (80%) ↑ 500 µM (148%)	[20]
*Oryza sativa* L. (ZJ 88) *Oryza sativa* L. (XS 134)	2,4-D 0.8 kg a.i. ha^−1^	15 days	Roots	CAT ↑ 0.8 kg a.i. ha^−1^ (15%); SOD ↑ 0.8 kg a.i. ha^−1^ (79%); APX ↑ 0.8 kg a.i. ha^−1^ (15%); POD ↓ 0.8 kg a.i. ha^−1^ (7%); CAT ↑ 0.8 kg a.i. ha^−1^ (19%); SOD ↑ 0.8 kg a.i. ha^−1^ (32%); APX ↑ 0.8 kg a.i. ha^−1^ (54%); POD ↑ 0.8 kg a.i. ha^−1^ (2%);	H_2_O_2_ ↑ 0.8 kg a.i. ha^−1^ (59%) O_2_^•−^ ↑ 0.8 kg a.i. ha^−1^ (29%) H_2_O_2_ ↑ 0.8 kg a.i. ha^−1^ (22%) O_2_^•−^ ↑ 0.8 kg a.i. ha^−1^ (19%)	↑ 0.8 kg a.i. ha^−1^ (214%) ↑ 0.8 kg a.i. ha^−1^ (121%)	[57]
*Brassica napus* L.	Metazachlor 0, 0.2, 0.4 mM	14 days 28 days	Leaves	CAT ↑ 0.2 Mm (80%); ↑ 0.4 mM (25%); SOD ↑ 0.2 Mm (30%); ↑ 0.4 mM (25%); APX ↑ 0.2 Mm (42%); ↑ 0.4 mM (35%); POD ↑ 0.2 Mm (170%); ↑ 0.4 mM (130%); GR ↑ 0.2 Mm (42%); ↑ 0.4 mM (83%); CAT ↑ 0.2 Mm (107%); ↑ 0.4 mM (175%); SOD ↑ 0.2 Mm (15%); ↑ 0.4 mM (68%); APX ↑ 0.2 Mm (42%); ↑ 0.4 mM (65%); POD ↑ 0.2 Mm (22%); ↑ 0.4 mM (220%); GR ↑ 0.2 Mm (30%); ↑ 0.4 mM (63%);	nd	↑ 0.2 mM (10%) ↑ 0.4 mM (23%) ↑ 0.2 mM (40%) ↑ 0.4 mM (43%)	[58]
*Setaria italica* L. (Jingu 21) *Setaria italica* L. (Zhangzagu 3) *Setaria italica* L. (Zhangzagu 5) *Setaria italica* L. (Zhangzagu 10)	Fluroxypyr 0, 0.5, 1, 2, 4 L a.i. ha^−1^	15 days	Leaves	CAT ↑ 0.5 L a.i. ha^−1^ (138%); ↑ 1 L a.i. ha^−1^ (480%); ↑ 2 L a.i. ha^−1^ (265%); ↑ 4 L a.i. ha^−1^ (65%); SOD ↑ 0.5 L a.i. ha^−1^ (75%); ↑ 1 L a.i. ha^−1^ (98); ↑ 2 L a.i. ha^−1^ (75%); ↑ 4 L a.i. ha^−1^ (75%;); APX ↑ 0.5 L a.i. ha^−1^ (72%); ↑ 1 L a.i. ha^−1^ (300%); ↑ 2 L a.i. ha^−1^ (155%); ↑ 4 L a.i. ha^−1^ (163%); POD ↑ 0.5 L a.i. ha^−1^ (80%); ↑ 1 L a.i. ha^−1^ (213%); ↑ 2 L a.i. ha^−1^ (200%); ↑ 4 L a.i. ha^−1^ (185%); GR ↑ 0.5 L a.i. ha^−1^ (57%); ↑ 1 L a.i. ha^−1^ (255%); ↑ 2 L a.i. ha^−1^ (150%); ↑ 4 L a.i. ha^−1^ (100%); CAT ↑ 0.5 L a.i. ha^−1^ (110%); ↑ 1 L a.i. ha^−1^ (210%); ↑ 2 L a.i. ha^−1^ (222%); ↑ 4 L a.i. ha^−1^ (115%); SOD ↑ 0.5 L a.i. ha^−1^ (575%); ↑ 1 L a.i. ha^−1^ (673%); ↑ 2 L a.i. ha^−1^ (718%); ↑ 4 L a.i. ha^−1^ (520%); APX ↑ 0.5 L a.i. ha^−1^ (65%); ↑ 1 L a.i. ha^−1^ (212%); ↑ 2 L a.i. ha^−1^ (243%); ↑ 4 L a.i. ha^−1^ (118%); POD ↑ 0.5 L a.i. ha^−1^ (100%); ↑ 1 L a.i. ha^−1^ (110%); ↑ 2 L a.i. ha^−1^ (180%); ↑ 4 L a.i. ha^−1^ (185%); GR ↑ 0.5 L a.i. ha^−1^ (32%); ↑ 1 L a.i. ha^−1^ (142%); ↑ 2 L a.i. ha^−1^ (272%); ↑ 4 L a.i. ha^−1^ (97%); CAT ↑ 0.5 L a.i. ha^−1^ (412%); ↑ 1 L a.i. ha^−1^ (370%); ↑ 2 L a.i. ha^−1^ (311%); ↑ 4 L a.i. ha^−1^ (435%); SOD ↑ 0.5 L a.i. ha^−1^ (72%); ↑ 1 L a.i. ha^−1^ (140%); ↑ 2 L a.i. ha^−1^ (227%); ↑ 4 L a.i. ha^−1^ (125%); APX ↑ 0.5 L a.i. ha^−1^ (15%); ↑ 1 L a.i. ha^−1^ (20%); ↑ 2 L a.i. ha^−1^ (92%); ↑ 4 L a.i. ha^−1^ (70%); POD ↑ 0.5 L a.i. ha^−1^ (28%); ↑ 1 L a.i. ha^−1^ (83%); ↑ 2 L a.i. ha^−1^ (90%); ↑ 4 L a.i. ha^−1^ (125%); GR ↑ 0.5 L a.i. ha^−1^ (18%); ↑ 1 L a.i. ha^−1^ (105%); ↑ 2 L a.i. ha^−1^ (295%); ↑ 4 L a.i. ha^−1^ (25%); CAT ↑ 0.5 L a.i. ha^−1^ (293%); ↑ 1 L a.i. ha^−1^ (320%); ↑ 2 L a.i. ha^−1^ (430%); ↑ 4 L a.i. ha^−1^ (110%); SOD ↑ 0.5 L a.i. ha^−1^ (138%); ↑ 1 L a.i. ha^−1^ (202%); ↑ 2 L a.i. ha^−1^ (230%); ↑ 4 L a.i. ha^−1^ (140%); APX ↑ 0.5 L a.i. ha^−1^ (113%); ↑ 1 L a.i. ha^−1^ (163%); ↑ 2 L a.i. ha^−1^ (345%); ↑ 4 L a.i. ha^−1^ (235%); POD ↑ 0.5 L a.i. ha^−1^ (88%); ↑ 1 L a.i. ha^−1^ (235%); ↑ 2 L a.i. ha^−1^ (670%); ↑ 4 L a.i. ha^−1^ (740%); GR ↑ 0.5 L a.i. ha^−1^ (13%); ↑ 1 L a.i. ha^−1^ (62%); ↑ 2 L a.i. ha^−1^ (195%); ↑ 4 L a.i. ha^−1^ (109%);	H_2_O_2_ ↑ 0.5 L a.i. ha^−1^ (70%) ↑ 1 L a.i. ha^−1^ (130%) ↑ 2 L a.i. ha^−1^ (160%) ↑ 4 L a.i. ha^−1^ (182%) O_2_^•−^ ↑ 0.5 L a.i. ha^−1^ (3%) ↑ 1 L a.i. ha^−1^ (10%) ↑ 2 L a.i. ha^−1^ (15%) ↑ 4 L a.i. ha^−1^ (28%) H_2_O_2_ ↑ 0.5 L a.i. ha^−1^ (2%) ↑ 1 L a.i. ha^−1^ (10%) ↑ 2 L a.i. ha^−1^ (30%) ↑ 4 L a.i. ha^−1^ (60%) O_2_^•−^ ■ 0.5 L a.i. ha^−1^ (0%) ↑ 1 L a.i. ha^−1^ (2%) ↑ 2 L a.i. ha^−1^ (2%) ↑ 4 L a.i. ha^−1^ (3%) H_2_O_2_ ↑ 0.5 L a.i. ha^−1^ (42%) ↑ 1 L a.i. ha^−1^ (55%) ↑ 2 L a.i. ha^−1^ (55%) ↑ 4 L a.i. ha^−1^ (60%) O_2_^•−^ ■ 0.5 L a.i. ha^−1^ (0%) ■ 1 L a.i. ha^−1^ (0%) ■ 2 L a.i. ha^−1^ (0%) ↑ 4 L a.i. ha^−1^ (10%) H_2_O_2_ ↑ 0.5 L a.i. ha^−1^ (1%) ↑ 1 L a.i. ha^−1^ (12%) ↑ 2 L a.i. ha^−1^ (13%) ↑ 4 L a.i. ha^−1^ (80%) O_2_^•−^ ↑ 0.5 L a.i. ha^−1^ (5%) ↑ 1 L a.i. ha^−1^ (10%) ↑ 2 L a.i. ha^−1^ (10%) ↑ 4 L a.i. ha^−1^ (13%)	↑ 0.5 L a.i. ha^−^ (35%) ↑ 1 L a.i. ha^−1^ (52%) ↑ 2 L a.i. ha^−1^ (62%) ↑ 4 L a.i. ha^−1^ (80%) ↑ 0.5 L a.i. ha^−1^ (10%) ↑ 1 L a.i. ha^−1^ (37%) ↑ 2 L a.i. ha^−1^ (52%) ↑ 4 L a.i. ha^−1^ (65%) ■ 0.5 L a.i. ha^−1^ (0%) ↑ 1 L a.i. ha^−1^ (7%) ↑ 2 L a.i. ha^−1^ (20%) ↑ 4 L a.i. ha^−1^ (25%) ↑ 0.5 L a.i. ha^−1^ (20%) ↑ 1 L a.i. ha^−1^ (37%) ↑ 2 L a.i. ha^−1^ (65%) ↑ 4 L a.i. ha^−1^ (80%)	[59]

* Approximate percentage values relative to controls for antioxidants enzyme, ROS and lipid peroxidation; Lipid peroxidation—determined by measuring the concentration of malondialdehyde as thiobarbituric acid reactive substances; † Histochemical analysis of ROS; ↑ Increase in roots and leaves with the application of herbicide in concentrations cited; ↓ Decrease in roots and leaves with the application of herbicide in concentrations cited; ■ Unchanged; nd, not determined. SOD—superoxide dismutase; CAT—catalase; APX—ascorbate peroxidase; POD—peroxidase; GR—glutathione reductase; GST—glutathione-S-transferase; O_2_^•−^—superoxide radical; H_2_O_2_—hydrogen peroxide; ZJ0273—Propyl 4-(2-(4,6-dimethoxypyrimidin-2-yloxy)benzylamino)benzoate; 2,4-D—2,4-dichlorophenoxyacetic acid.

**Table 3:** Antioxidant-related genes differentially expressed identified from RNA-Seq studies performed for weed herbicide resistance investigation after herbicide treatment.

Weed Species	Herbicide Resistance	ROS Scavenging Pathway Genes	Reference
*Avena fatua*	Pinoxaden Flucarbazone	GST, SOD	[12]
*Alopecurus aequalis* Sobol	Mesosulfuron-methyl	GST, POD	[62]
*Apera spica-venti*	Sensitive	GST	[63]
*Brachypodium hybridum*	Pinoxaden	GST, POD	[64]
*Eleusine indica* L.	Glyphosate	GST	[65]
*Lolium spp.*	Pyroxsulam; Iodosulfuron+mesosulfuron	GST	[66]
*Beckmannia syzigachne*	Fenoxaprop-P-ethyl	GST, POD	[67]
*Descurainia sophia* L.	Tribenuron-methyl	GST, POD	[68]
*Alopecurus myosuroides* L.	Iodosulfuron+mesosulfuron	GST, POD	[69]
*Euphorbia esula*	Glyphosate	GST	[70]
*Eleusine indica* L.	Paraquat	GLR, MDAR, GR, POD, GST, CAT, Trx	[8]
*Lolium rigidum*	Diclofop-methyl	GST	[71]

GST—glutathione-S-transferase; POD—peroxidase; GLR—glutaredoxin; MDAR—monodehydroascorbate reductase; GR—glutathione reductase; CAT—catalase; Trx—thioredoxin.

## Abbreviations

^1^O_2_	singlet oxygen
O_2_^•−^	superoxide radical
H_2_O_2_	hydrogen peroxide
OH^•^	hydroxyl radical
SOD	superoxide dismutase
CAT	catalase
APX	ascorbate peroxidase
GPX	glutathione peroxidase
POD	peroxidase
PRX	peroxiredoxin
MDHAR DHAR	monodehydroascorbate reductase dehydroascorbate reductase
GR	glutathione reductase
GST	glutathione-S-transferase
ASC	ascorbic acid
GSH	glutathione
Trx	thioredoxin
